# Development of a maternal health summer program curriculum to expose high school students to pharmacy, environmental health science, and community health worker careers

**DOI:** 10.3389/fpubh.2026.1793225

**Published:** 2026-07-01

**Authors:** Grace Olorunyomi, Cecilia Torres, Jazmyne Jones, Kennedi Norwood, Kehinde Idowu, Kimberly Pounds, Denae King, Veronica Ajewole-Mwema, Ivy Poon, Esther Olaleye

**Affiliations:** 1 College of Pharmacy and Health Sciences, Texas Southern University, Houston, TX, United States; 2 Barbara Jordan-Mickey Leland School of Public Affairs, Texas Southern University, Houston, TX, United States

**Keywords:** career exploration, health workforce pipeline, high school research training, maternal health, maternal health education, public health

## Abstract

**Introduction:**

The State of Texas has the highest number of maternal deaths in America, and maternal health outcomes are worsened by a shortage of maternal health providers.

**Methods:**

We designed four training modules in pharmacy practice, environmental health science, and community health to train a cohort of high school students in a university-based summer research program. The training modules consisted of four self-paced online lessons, followed by a one-month period of small-group, hands-on research projects guided by research staff, and a cumulative poster presentation. The training was offered as part of the 8-week Maternal Health High School Summer Research Program at Texas Southern University. We assessed students’ knowledge through quiz scores and student surveys.

**Results:**

20 students participated, of whom 19 completed the modules. The report indicated that students’ engagement and performance were high across the four lessons, with participation ranging from 83 to 100%, and participants correctly answering 80 to 97% of the questions. Student feedback further indicated that the program supported early-career exploration, increasing students’ interest in maternal and child health and healthcare-related career pathways.

**Discussion:**

Our experience demonstrates a model for introducing health-related disciplines to high school students early in their career exploration years. Early education on maternal health may increase awareness of public health issues and encourage exploration of careers in public health and maternal health.

## 1 Introduction

Maternal mortality is a critical health problem in the United States (U. S.). From 2000 to 2020, the maternal mortality ratio (MMR), defined as the number of maternal deaths per 100,000 live births, increased from 12 to 20 in North America (1). The U. S. experienced the greatest increase in MMR from 2000 to 2015 among all countries worldwide. In the U. S, Texas is one of the top states with a pregnancy-related death rate of 38.1 per 100,000 births compared to the U. S. average of 26.3 per 100,000 live births (2). According to the March of Dimes, in 2023, 22.0 and 10.8% of live births in Texas were to women who received inadequate prenatal care and late or no prenatal care, respectively (3). Notably, Texas ranks among the lowest in state health system performance (50 out of 51) (4), highlighting the need to improve health care access, prevention, and treatment, as well as the health outcomes of pregnant women.

Expanding the perinatal workforce has been identified as a key strategic goal in Texas and nationally (5, 6). According to the Texas OB-GYN Workforce Study, a major challenge is the shortage of obstetricians and gynecologists (OB-GYNs) across the state (7). Texas is also projected to experience a substantial nursing shortage in the coming decade, with demand for registered nurses (RNs) expected to exceed supply by more than 57,000 positions, compounded by rising vacancy rates among both RNs and licensed vocational nurses (LVNs) (8). Workforce studies have also identified shortages in midwifery care providers in Texas, with documented deficits of certified nurse-midwives in medically underserved areas (9). As the remaining OB-GYN workforce continues to face increasing demand, opportunities emerge for other health professionals (e.g., pharmacists) and community-based roles such as Community Health Workers (CHWs) to expand their scope in bridging gaps in essential maternal care and support (10, 11). In 2024, Texas further advanced this approach through the passage of House Bill 1,575, which allows doulas to become CHWs and enroll as a new provider type under the Medicaid Care Management for Children and Pregnant Women (CPW) program (12). These developments reflect broader workforce trends emphasizing the expansion of allied and community-based professionals to support maternal health amid persistent clinician shortages.

Aligned with the overall goal of improving maternal health outcomes in Texas, the Gulf Coast Collaborative Center for Maternal Health Research, Education, Advanced Training, Community Engagement, and Health Empowerment (MHREACH) program was established at Texas Southern University (TSU) in 2023. Details about the MHREACH and the first iteration of the MHREACH Maternal Health Education and Research Training (MHERT) program in 2024 have been published (13). The MHERT program was designed for high school students recruited from the Greater Houston Communities. The goal of the MHERT program is to provide early exposure to high school students in public health, research, and career pathways in the health professions.” This report describes the development, implementation, and assessment of four new modules focusing on cardiovascular disease, mental health, environmental health, and contextual factors affecting health in the summer of 2025.

## 2 Pedagogy

Learning is a process in which a student’s knowledge increases over time as they reflect on prior experiences, develop motivation to learn, and achieve mastery, ultimately becoming a self-directed learner (14). We intentionally sequenced the students’ learning experience by administering a pre-assessment survey to reflect on their prior experience and motivation toward learning maternal health topics, followed by a series of online modules with assessment quizzes to ensure mastery of the topics. We recognize that high school students may lack prior knowledge of pathophysiology, clinical diagnosis, and epidemiology, and mastering these competencies can be a significant challenge. Therefore, we allowed multiple attempts to complete the lessons. Additionally, the course was structured with consistency and introduced desirable difficulties by assigning a hands-on project that required integrating clinical, environmental health science, and public health concepts after completing the online lessons (15). From learning how to write without plagiarizing to synthesizing study findings and creating a poster presentation, each learner gains knowledge through lower complexity Bloom’s taxonomy skills (knowledge) and progresses to a deeper degree of learning (application and synthesis) (16). We also engage the students in tracking progress and reflection through a post-assessment survey.

## 3 Learning environment and curriculum development

The investigator team participated in a team meeting to reflect on the first iteration of MHERT and began reviewing the learning outcomes and assessment data of the 2024 summer MHERT high school student cohort (*n* = 10) in February 2025 (13). The team reviewed the syllabus, delivery methods, topics covered, course performance, pre- and post-training surveys, and student feedback. This process yielded several important observations.

First, student reflections suggested that interest in learning about the topics was motivated by family-related maternal health experiences, aligning with principles of Social Cognitive Career Theory related to career exploration (17). Second, the first-year cohort provided positive feedback and completed the lessons with ease (100% completed on time) in the structured 8-week program, which consisted of 4 weeks of independent pre-recorded online training modules in the NearPod learning platform with weekly check-ins, followed by 4 weeks of hands-on research experience guided by one MHREACH research staff, one part-time learning specialist, and one senior faculty (18). Third, the high school trainees reported a variety of career interests, including basic sciences, public health, pharmacy, and undecided, in the pre-assessment surveys. Fourth, the program review identified the need to expand and update curricula to better align with current maternal health issues and priorities.

Based on the observations above, the MHERT research team discussed and decided to maintain the recruitment strategies described in our prior publication (13). In this second iteration, eligibility criteria were expanded from students entering the 11th and 12th grades to include students in grades 8 through 12. Participants were recruited through flyers sent to high school counselors and principals in Greater Houston–area high schools, and through outreach at community events. Limiting recruitment to the Greater Houston area was intentional and aligned with our center’s mission, which is to address maternal health problems in Texas with a particular focus on local communities. As such, recruiting students from the Houston area allows us to engage participants who are embedded in and can directly contribute to the communities most impacted. All eligible students were encouraged to apply, and applicants were selected based on academic merit, performance, and letters of recommendation. Eligibility required a commitment of approximately 8 h per week for the 8-week summer program (approximately 64 h to complete). Prior to acceptance, all applicants are informed of the program expectations, including the anticipated time commitment, allowing them to determine whether they can balance participation with other obligations such as summer employment or concurrent programs. The estimated 8 h per week represents a conservative high estimate. As the didactic content is delivered through pre-recorded, asynchronous modules, students can progress at their own pace and are also allowed to revisit lectures and repeat quizzes until achieving the required benchmarks for the program (100% participation in lessons as tracked by NearPod and ≥ 80% for the quizzes), which means the actual time commitment may vary across participants.

A summary of recruitment strategies and selection criteria is provided in Table 1.

**Table 1:** Summary of participant recruitment and selection criteria.

Category	Description
Recruitment strategies	Flyers distributed to Greater Houston-area high schools’ counselors and principals; outreach conducted at community events
Target population	High school students entering grades 8–12
Eligibility criteria	Enrollment in high school (grades 8–12)
Program commitment	8 h per week for 8 weeks (~64 total hours)
Selection criteria	Academic performance (GPA/grades) and letters of recommendation

The original program structure, which involves utilizing independent online training modules followed by hands-on research experience, was also maintained (13). The didactic portion of the program was delivered entirely in a virtual, asynchronous format. Modules were pre-recorded in NearPod, allowing for self-paced learning so students can engage with the material at times convenient to their schedules. The total time commitment to complete the instructional modules was estimated at a conservative 8 h per week, though actual time varied by individual learning pace. The actual recording length of each module is up to 2 h.

The hands-on research component required students to actively participate rather than observe. It may incorporate either virtual or in-person elements, depending on the project. Students worked in small groups (3–4 participants) with assigned mentors, and meeting times were scheduled based on mutual availability. Research activities varied by group: some students engaged in community-based work (e.g., conducting qualitative interviews), while others conducted their work entirely online (quantitative data analysis and analyzing focus group data). While not all experiences were in person, all projects were designed to provide applied, practical research experience. Overall, the hybrid structure, combining asynchronous instruction with mentor-guided, flexible research activities, was intended to provide meaningful, hands-on learning opportunities adaptable to different project types.

To increase exposure to a variety of career fields with our additional expertise, we decided to create four new modules: (1) cardiovascular disease in pregnancy, (2) mental health in pregnancy, (3) environmental exposures and health in pregnancy, and (4) contextual factors in health and community support in pregnancy. These topics covered the top causes of maternal mortality and morbidity in Texas, environmental influences on health and prevention strategies, and expanding the use of CHWs and doulas to improve maternal care, which is a key recommendation in the Texas Maternal Mortality and Morbidity Review Committee (MMMRC) report (6). Additionally, we increased the number of students from 10 to 20 to reflect our increased program staff capacity. The learning objectives of the new modules are described in Table 2. Students who fulfilled all program requirements each received a $250 stipend, along with additional incentives, including a backpack, folder, notepad, pen, glass water bottle, portable fan, first aid kit, hand sanitizer, earbuds, an MHREACH T-shirt, and an MHREACH lab coat, all provided through the HRSA-funded Maternal Health Research Center (MHREACH).

**Table 2:** Modules lesson plan and objectives.

Lesson	Topics	Instructor discipline	Lesson objectives	Assessment methods
1	Cardiovascular Diseases in Pregnancy	Pharmacy	Understand the pathophysiology of cardiovascular diseases in Pregnancy. Describe the conditions (e.g., hypertensive disorders in pregnancy, arrhythmias in pregnancy, venous thromboembolism), risk factors, and clinical presentations. Discuss early detection and prevention strategies.	Three multiple-choice questions One matching pair exercise Write a reflection after watching a video of a mother’s experience with preeclampsia.
2	Mental Health in Pregnancy	Pharmacy	Understand what perinatal depression, bipolar disorder, perinatal anxiety, and postpartum psychosis are. Recognize signs and symptoms of these conditions. Understand the importance of support, treatment, and reducing stigma. Reflect on how they can help others and seek help themselves if needed.	Two multiple-choice questions One open response question to list ways to support someone with a perinatal mental health condition. Write a reflection after watching a video of a mother’s experience with postpartum depression and obsessive-compulsive disorder.
3	Environmental Exposures and Health in Pregnancy	Environmental Toxicology	Define environmental exposures and give examples. Describe how substances like lead, PFAS, and microplastics can enter the body. Explain how these exposures can affect maternal and fetal health. Understand basic mechanisms of harm during pregnancy. Identify ways to reduce or prevent harmful exposures.	One open-response question to gain an understanding of environmental exposure. Two multiple-choice questions. Write a reflection after watching two videos: a case study on how environmental exposures affect public health and a recent policy change in regulating environmental toxins. One matching pair game.
4	Contextual Factors in Health and Community Support in Pregnancy	Public Health	Understand the roles and responsibilities of CHWs. Learn about maternal health challenges in marginalized communities. Explore how CHWs and other health professionals work together to support and improve maternal health.	Two multiple-choice questions. One drag-and-drop activity to match CHW action to maternal community needs. One poll to gain an understanding of whether learners have known someone who is a CHW. One collaborative board to share their desire to be a CHW and potential serving area interests.

### 3.1 Instructional strategies

Instructional strategies across the lessons emphasized active, learner-centered, and age-appropriate approaches to support understanding, engagement, and application of knowledge among high school students. The curriculum integrated current, relevant, and evidence-based content delivered through online lectures that incorporated interactive elements, including knowledge checks, reflective prompts, scenario-based activities, visual learning resources, real-world examples, and lived-experience narratives to simplify abstract and complex concepts in practical contexts. To ensure understanding across age groups and address variation in developmental stages across grades 8–12, all instructional materials were developed at an 8th-grade reading level, with an emphasis on clear, simplified explanations that maintain learners’ attention. The curriculum prioritized foundational public health concepts understandable without prior knowledge of anatomy or physiology, allowing students of varying academic backgrounds to engage effectively. Instructional strategies were designed to support different learning paces while maintaining consistent expectations for all participants. Collectively, these strategies were designed to promote foundational understanding, critical thinking, and the relevance of maternal health topics to students’ lives and future career interests.

#### 3.1.1 Cardiovascular health in pregnancy

Cardiac events accounted for 17% of linked maternal deaths in Texas from 2016 to 2019 and 15% from 2020 to 2023, ranking as the leading and second leading causes during these respective periods (19). In response to this critical public health issue, we designed a lesson to introduce learners to foundational knowledge of cardiovascular disorders in pregnancy, highlighting how pregnancy affects the cardiovascular system and common cardiac conditions associated with it. The learning objectives included defining cardiovascular disorders, describing how pregnancy affects the heart and blood vessels, identifying common cardiovascular conditions that occur during pregnancy, recognizing warning signs, and understanding the importance of early detection and management. The lesson addressed common cardiovascular conditions encountered during pregnancy, including hypertensive disorders of pregnancy (hypertension, preeclampsia, and eclampsia), hypercholesterolemia, arrhythmias, and venous thromboembolism. Topics were selected based on a review published in the American Heart Association’s *Circulation* and were adapted for a high school audience (20). All educational materials were developed in alignment with established clinical and therapeutic guidelines for each condition. The content integrated foundational concepts with current clinical guidance and a lived-experience narrative example to illustrate cardiovascular risks during pregnancy. Lecture slides incorporated visual aids, including illustrated diagrams and visual summaries, to support understanding of cardiovascular changes and conditions during pregnancy. Learner understanding was assessed through knowledge checks, including matching exercises, multiple-choice questions, and a short, reflective, open-ended prompt. Collectively, these components supported students’ foundational understanding of cardiovascular health in pregnancy and emphasized its relevance to maternal health outcomes.

#### 3.1.2 Mental health in pregnancy

Mental health conditions are an important contributor to maternal morbidity and mortality, with suicide accounting for 14% of linked maternal deaths in Texas from 2016 to 2019 and 9% from 2020 to 2023 (19). In response, we developed a lesson focused on mental health during and after pregnancy, with the goal of increasing understanding of the impact of mental health conditions during pregnancy and introducing strategies for recognizing and supporting individuals experiencing these conditions. Learning objectives included defining mental health conditions, identifying common perinatal mental health conditions, recognizing signs and symptoms, understanding the importance of treatment, support, and stigma reduction, and reflecting on how to support others and seek help when needed. The lesson emphasized perinatal mental health conditions, including depression, anxiety and anxiety-related disorders, bipolar disorder, and postpartum psychosis. Content selection was guided by the American College of Obstetricians and Gynecologists (ACOG) Clinical Practice Guidelines on Mental Health Conditions During Pregnancy and Postpartum, and all educational materials were developed in accordance with these guidelines (14). Instructional content incorporated current information, relevant statistics, and available support resources. Visual aids, including conceptual diagrams of perinatal mental health risk factors, visual summaries, a short video on lived experience, and resource listings, were used to illustrate how mental health conditions may present during pregnancy and the postpartum period. Assessment of learner understanding included multiple-choice questions, open-ended responses, and scenario-based questions. Overall, this lesson aimed to build foundational knowledge while promoting awareness of perinatal mental health and the importance of seeking support.

#### 3.1.3 Environmental health and pregnancy

Emerging evidence shows that exposure to toxic environmental agents such as chemicals, air pollution, and climate-related factors before and during pregnancy is linked to adverse birth, maternal, and fetal health outcomes, including miscarriage, preterm birth, low birth weight, and neurodevelopmental disorders (15). In response to this growing evidence, we designed a lesson on environmental exposures and maternal health to introduce learners to the impact of environmental agents on birth and pregnancy outcomes. The learning objectives include defining environmental exposures and providing examples, describing how environmental agents such as lead, PFAS, pesticides, and microplastics enter the body, and explaining their impact on maternal and fetal health. Learners were also introduced to the basic mechanisms of harm during pregnancy and strategies to reduce or prevent harmful exposures. The lecture covered key concepts, including common environmental pollutants, exposure pathways, pregnancy and sensitivity, and the mechanisms of action of these agents. It integrates established information, current scientific research, and real-world case studies to build foundational knowledge and help learners identify environmental risks and understand their impacts on maternal and public health. The lecture slides included a variety of visual learning resources, including infographics, conceptual diagrams, news articles, and short videos on historical environmental exposure events, to make the content more engaging and simplify complex scientific concepts. Assessment methods included true/false questions, matching pairs, and open-ended questions to gage students’ understanding and retention. Altogether, the components of this lecture ensured that learners gained a clear, fundamental understanding of environmental exposures and their adverse effects on maternal and child health, highlighting the importance of the topic.

#### 3.1.4 Community health and support in pregnancy

The Texas Maternal Mortality and Morbidity Review Committee and the Department of State Health Services’ (MMMRC-DHSH) Joint Biennial 2024 Report recommended increased engagement of community-based support roles, such as CHWs, to address gaps in perinatal care and improve maternal health outcomes (6). CHWs play a crucial role in facilitating care coordination and enhancing connections to perinatal services and nutritional programs, serving as an integral link within the healthcare system (6). Many families face challenges related to navigating prenatal care, nutrition programs, and social services, which can negatively affect maternal health outcomes. Additional contextual factors, such as living conditions, socioeconomic status, and limited access to education, may further complicate the process of identifying available services. Accordingly, we developed a lesson to highlight the role of CHWs in providing support during and after pregnancy and to introduce learners to contextual factors that may influence maternal health outcomes. The learning objectives included understanding the role of CHWs, explaining how CHWs collaborate with other healthcare professionals (e.g., doulas, social workers, counselors, and obstetricians), and identifying social and environmental factors that may contribute to maternal health challenges. The lesson emphasized how CHWs serve as trusted links between communities and healthcare systems, facilitating care navigation and connections to services and resources. The Learning strategies incorporated interactive components to promote engagement and knowledge application, including pre- and post-lesson multiple-choice quizzes, polls, and drag-and-drop activities designed to assess learners’ ability to distinguish between community needs and CHW responsibilities. Examples highlighting MHREACH community engagement efforts and their impact, along with discussion board prompts used as a scenario-based activity, allowed learners to explore how CHWs apply these concepts in practice and encouraged reflection on the role of CHWs and the importance of community-based support in maternal health. Together, these activities strengthened students’ understanding of community health roles and highlighted the importance of coordinated support systems during pregnancy.

In addition to the four new lessons, the students also completed six other lessons: plagiarism training, maternal health epidemiology, causes and solutions to address maternal health problems, common research methods, CITI training on human subject research, how to conduct a literature review, and artificial intelligence/machine learning in research. Details about these lessons are described in a previous publication (13).

### 3.2 Assessment

Our program staff tracked our students’ progress weekly during the first 4 weeks of independent online modules. A progress chart was posted weekly, every Friday, on our Google Classroom site, showing learners’ names and progress in each module (complete, in progress, or not started). A completion grade was defined as a full participation score in each Nearpod lesson and a score of 80% or higher on the post-lesson quiz. Multiple attempts were allowed, and the highest score attempt was used to track students’ progress. Email reminders were sent to students and parents of those making unsatisfactory progress.

Each NearPod online lesson includes learning objectives, a recorded presentation, and a variety of assessment activities, such as a knowledge check quiz, reflection, matching game, and collaboration board. The program staff reviewed the lesson report weekly to update the progress chart.

Students completed a pre- and post-training survey that included six open-ended questions to gather information about their career plans, potential college majors, rationale for applying to the program, and previous training in maternal health. Students were also encouraged to complete voluntary mid-course and final evaluation surveys to provide feedback about the training program. The survey instrument was reviewed and approved by the TSU Institutional Review Board (IRB), and all students completed the informed consent process.

At the end of the 8-week MHERT program, students created and presented a professional poster at the Texas Southern University Leadership, Education, and Advancement in Undergraduate Research Pathways (TSU–LEAP) Program Summit. The TSU-LEAP Summit was open to faculty, research staff, undergraduate, and graduate students, creating an opportunity for high school students to learn about college-level research projects and receive feedback on their own research projects from the broader TSU research community.

## 4 Results and assessments

### 4.1 Student demographics

We received 36 applications for the program. The program staff reviewed each application. Of the 36 applicants, we admitted and enrolled 20 students into the program. One student dropped out of the program due to personal circumstances, and therefore, a total of 19 high school students completed the training. Detailed demographic information for student participants is shown in Table 3. Race and ethnicity were collected using standardized Office of Management and Budget (OMB) categories and more subgroup data (e.g., within the “Asian” category) was not collected (21). Additionally, 6 of the 10 high school students who were trained in the summer of 2024 applied to return as peer mentors. Two out of those six served as peer mentors for the 2025 cohort.

**Table 3:** Student demographics (*n* = 19).

Characteristics	Category	Number (Percentage)
Age range, n (%)	13–15	5 (26.32%)
	16–17	13 (68.42%)
	18–19	1 (5.26%)
Gender, n (%)	Female	14 (73.68%)
	Male	5 (26.32%)
Race & ethnicity, n (%)	Asian	14 (73.68%)
	Black or African American	4 (21.05%)
	White	0 (0.00%)
	Hispanic	1 (5.26%)
Types of high school, n (%)	Public high school (ISD)	14 (73.68%)
	Charter school	4 (21.05%)
	Private school	1 (5.26%)
Location of high school^a^, n (%)	City	6 (31.58%)
	Suburban	12 (63.16%)
	Rural	1 (5.26%)

a Location of high school based on the National Center for Education Statistics (NCES) (25).

### 4.2 Online lesson results

**Figure 1:**
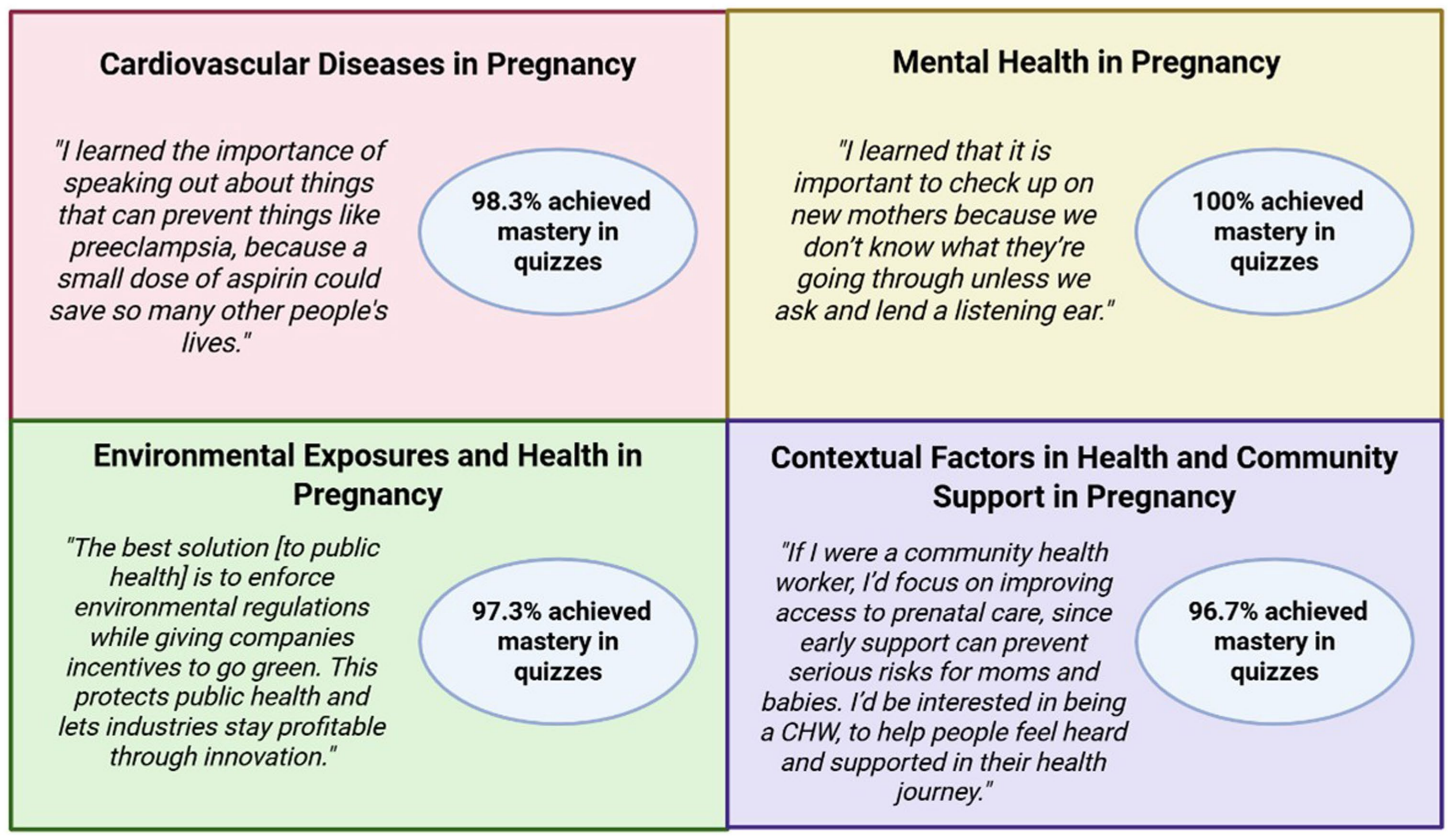
Student performance metrics and reflections on lessons.

The enrolled students successfully completed the online learning lessons in the first 4 weeks of the program. A summary of their performance in the newly developed lessons is demonstrated in Figure 1. Overall, the students demonstrated measurable knowledge gains and interest in the topics, as reflected in their quiz performance and open-ended responses. The report indicated high levels of student engagement and performance across the four lessons, with participation rates ranging from 83 to 100% and participants’ correct response rates ranging from 80 to 97%. All participants successfully met the program requirements and achieved the required benchmark performance on assessments.

### 4.3 Hands-on research projects

Upon completion of all online lessons, students were asked to rank their interests in participating in a hands-on research project in the following areas: analysis of secondary data on a maternal health topic, analysis of secondary data on PFAS and folate in pregnancy, focus group analysis on hypertension in pregnancy, and a community project in creating a teen pregnancy resource guide. Based on their preferences and completion of the online lessons, the research staff assigned each student to a group of 3 to 5 for a mentored research project over the next 4 weeks. Details about the projects are shown in Table 3.

### 4.4 Pre- and post-training surveys

#### 4.4.1 Pre-training survey

A total of 20 students completed the pre-training surveys. All students reported that the MHERT was their first training on maternal health topics. Five students reported having previous experience in research projects. In an open-response question, the majority of students (*n* = 12) declared they would pursue a career in medicine, followed by undecided (*n* = 6), allied health (*n* = 4), public health (*n* = 1), and business (*n* = 1; Table 4).

**Table 4:** Hands-on group projects (*N* = 19).

Project title	Mentor expertise	Research methods	Outcomes
Developing a Teen Parent Resource Guide	Public health (KN^a^, KP^a^)	Community Engaged Participatory Research	The students (*n* = 3) interviewed community members to gain feedback and created a Teen Parent Resource Guide.
Analyzing Maternal Health Focus Group Data	Pharmacy (JJ^a^)	Qualitative Analysis	The students (*n* = 4) reviewed focus group transcripts and used thematic analysis to find common themes.
Descriptive Analysis of Blood Pressure in Pregnancy	Epidemiology (CT^a^)	Analysis of Secondary Data	The students (*n* = 5; including one peer mentor) utilized the CDC^b^ NHANES^c^ dataset to identify demographic variables associated with high blood pressure during pregnancy.
Descriptive Analysis of Diabetes in Pregnancy	Epidemiology (CT)	Analysis of Secondary Data	The students (*n* = 5); including one peer mentor) utilized the CDC NHANES dataset to identify demographic variables that were associated with diabetes in pregnancy.
Exploring the link between PFAS Exposure and Folate Levels in Pregnant Women	Environmental Toxicology (GO^a^)	Analysis of Secondary Data	The students (*n* = 4) used the CDC NHANES dataset to investigate whether PFAS exposure was associated with folate levels during pregnancy.

a Author’s initials.

b CDC, Center for Disease Control.

c NHANES, National Health and Nutrition Examination Survey.

When asked what prompted them to apply for the training program, the majority indicated that they wanted to learn about research design and methods. A few excerpts highlighting this theme are listed below:

*“I joined this training program to gain research experience and learn the processes involved in creating research posters.”*

*“I saw this as a great opportunity to broaden my understanding of women’s health while also gaining insight into the research process and everything that goes into conducting meaningful studies.”*

*“I always had a dream of being part of a research program. I feel that it will allow me to get a grasp of what medical professionals in the real world deal with and help me to work with my peers to strengthen my skills. I am also intrigued by the topic of maternal health.”*

Several students expressed their interest in enrolling in the program was influenced by a family experience. A few excerpts highlighting this theme are listed below:

*“My stepmom is pregnant, and I would like to know more about maternal health so that I can understand for her and for whenever I have a family.”*

*“I wanted to apply to this training program to get more experience in research in a field I really cared about. My mom is in the maternal health field, and I was inspired by all that she does and how she is able to help so many women. Even though that I don’t plan to go to the pre-med route, I think this course will still be very helpful.”*

A few students expressed interest in enrolling in this training program to help others and contribute to improving human health.

*“I’ve read accounts where maternal health has been neglected in hospital settings and I believe I should educate myself on this topic as an aspiring physician.”*

*“In this course, I would hope to gain more insights through research that would allow me to be able to offer more holistic care to both the mother and the child.”*

*“I hope this course helps me explore the bigger picture of maternal health and how we can make it safer for everyone.”*

#### 4.4.2 Post-training survey

A post-training survey was distributed to students upon completion of the program. A total of 14 students consented to complete the post-training survey. When asked how the MHERT program has helped toward identifying a career path, here are a few highlights:

*“The program has helped me towards identifying my career path by providing me with exposure to different careers and fields I did not know existed.”*

*“I think the MHREACH program has been a major identifier for my future career path. Learning more about maternal health has allowed me to really understand lack of support mothers have and the program elevated my interest in healthcare. I think it’s also helped me realize I would feel fulfilled with a career in healthcare.”*

*“The MHREACH program really helped a lot with identifying my future career path because it introduced me to many faculty members and graduate students pursuing many of the things that were interesting to me. For me specifically, hearing from a practicing pharmacist as well as pharmacy students solidified my desire to go into pharmacy because even though it was something I was considering, I wasn’t sure how attainable it was until I met all these accomplished people in that field.”*

When asked about which study areas (e.g., clinical, public health, and environmental toxicology) were perceived as most interesting to the students, most students expressed public health (*n* = 8), followed by clinical (*n* = 5), and environmental toxicology (*n* = 1).

When asked about how they think the research training has helped with their professional growth, here are a few excerpts to highlight:

*“I didn’t realize how easy plagiarism can occur, even when you think you’re properly paraphrasing.”*

*“I was able to enhance my research skills through this project. I was introduced to Excel functions which help organize data and create pivot tables which I had not previously done before.”*

*“The research training has greatly contributed to my professional growth by strengthening my analytical thinking, problem-solving skills, and attention to detail. I have learned how to design studies, collect and interpret data, and communicate findings effectively, skills that are transferable to any scientific or professional setting. It has also improved my ability to work independently while collaborating as part of a research team, which has enhanced my confidence in contributing to larger projects. Overall, I think it prepared me to take on larger roles in research and build up my resume.”*

## 5 Discussion and lessons learned

High school represents a critical period for career exploration, as students begin identifying potential fields of study when applying to college. These years help students identify their first- and second-choice majors when applying to college. In this program, exposure to multiple disciplines, including epidemiology, pharmacy, environmental toxicology, and community health, was associated with increased interest in public health careers. While only 1 out of 20 students expressed interest in public health in the pre-assessment, 8 out of 14 students identified public health topics as most interesting in the post-assessment. This shift may be explained by the curriculum’s focus on teaching the multiple levels and domains that influence maternal health outcomes (16). This approach highlighted the broad relevance and applicability of public health concepts. It is our goal that students graduating from our program understand that solving maternal health problems requires a multidisciplinary team.

It is our goal that students graduating from our program understand that solving maternal health problems requires a multidisciplinary team. A key lesson learned is the importance of introducing students to the multidisciplinary nature of maternal health. The curriculum emphasized that improving maternal health outcomes requires collaboration among diverse professionals, including clinicians, allied health providers, basic scientists, community health workers, public health practitioners, and policymakers. This framing helped students understand the wide range of career pathways and roles involved in addressing complex health and public health challenges.

The program’s online format was a notable strength, as it allowed for flexible scheduling during the summer and reduced transportation-related challenges for students and families. Interactive online lessons, combined with structured reflection activities, supported deeper engagement and reinforced learning. Additionally, incorporating a hands-on project (active participation rather than observation) provided students with opportunities to apply the concepts they had learned, thereby promoting knowledge retention and critical thinking (22–24). The online lessons encourage structured reflections on lessons learned, promoting deeper engagement and a better understanding of the topics.

Although the program provided students with valuable knowledge and career preparation, the program team recognized that completing all requirements represented a substantial time commitment, particularly during the summer when many peers were on break. To support participation and sustained engagement, students were provided with a stipend and additional incentives. These incentives were intended to complement the program experience by recognizing students’ time and commitment while also supporting their academic and career development through early exposure to research, college-readiness, and opportunities for mentorship. The end-of-program symposium further enhanced the learning experience by allowing students to present their work and engage with university faculty and peers, fostering a sense of accomplishment and academic connection.

Finally, analysis of student reflections suggested meaningful and sustained learning, as participants connected course content to their personal and community experiences, including reflections on maternal health within their own families and environments (17). The hands-on project also served as an active learning strategy, enabling students to revisit, retrieve, and apply concepts introduced in the online lessons to their projects (22–24). This highlights the value of contextualized learning in enhancing relevance and promoting deeper understanding.

## 6 Limitations

One challenge we experienced was communication during the hands-on project in the latter 4 weeks of the program. Since we decided to restrict communication to emails, direct phone calls, and virtual meetings, we found that a few students were initially difficult to reach. Another limitation is that the number of questions required to access the knowledge is small, which limits engagement and interest and increases the learner burden of completing the course, making it inappropriate for their learning levels. Additionally, the cohort composition may not fully reflect the demographic distribution of the broader Houston student population, as it may be influenced by variation in the applicant pool and by the program’s small cohort size and by the lack of subgroup or sub-classification information for race categories.

## Data Availability

The original contributions presented in the study are included in the article/supplementary material, further inquiries can be directed to the corresponding author.
